# *In vitro* inhibitory effect of lysionotin on the activity of cytochrome P450 enzymes

**DOI:** 10.1080/13880209.2020.1787468

**Published:** 2020-07-16

**Authors:** Yang Li, Jing Qin, Hong Wu, Yongmei Xu, Li Zhang, Keren Su, Ying Cui, Haiping Wang

**Affiliations:** a Department of Neurology, The Affiliated Hospital of Qingdao University, Qingdao, China; b Department of Neurology, Zibo No. 4 People’s Hospital, Zibo, China; c Department of Laboratory, Yidu Central Hospital of Weifang, Weifang, China; d Department of Oncology, Binzhou Medical University Hospital, Binzhou, China; e Department of Cardiology, Shanxian Central Hospital, Heze, China; f Department of Pharmacy, Shanxian Central Hospital, Heze, China; g Department of Hematology and Nephrology, Shanxian Central Hospital, Heze, China

**Keywords:** CYP3A4, CYP2C19, CYP2C8, drug-drug interaction

## Abstract

**Context:**

Lysionotin, a major extraction of *Lysionotus pauciflorus* Maxim (Gesneriaceae), has a variety of pharmacological properties commonly used in the treatment of lung disease. A study of lysionotin on the activity of human liver cytochrome P450 (CYP) enzymes can provide guidance on the clinical application of lysionotin.

**Objective:**

This study investigated the interaction between lysionotin and CYPs.

**Material and method:**

The effects of 100 μM lysionotin on eight human liver CYP isoforms (i.e., 1A2, 3A4, 2A6, 2E1, 2D6, 2C9, 2C19 and 2C8) were investigated *in vitro* using human liver microsomes (HLMs) with specific inhibitor as positive control and untreated HLMs as control. Meanwhile, the enzyme kinetic parameters were calculated. A time-dependent study was performed with a time interval of 5 min in 30 min.

**Results:**

Lysionotin was found to inhibit the activity of CYP3A4, 2C19, and 2C8, with IC_50_ values of 13.85, 24.95, and 30.05 μM, respectively. The inhibition of CYP3A4 was performed in a non-competitive manner with the *Ki* value of 6.83 μM, while the inhibition of CYP2C19 and 2C8 was performed in a competitive manner with *Ki* values of 12.41 and 14.51 μM. Moreover, it was found that the inhibition of CYP3A4 was time-dependent with *K*_I_/*K*_inact_ value of 6.618/0.048 min/μM.

**Discussion and conclusions:** The *in vitro* inhibitory effect of lysionotin on the activity of CYP3A4, 2C19, and 2C8 indicated potential drug interactions between lysionotin and drugs metabolised by CYP3A4, 2C19, and 2C8. Further *in vivo* experiments are needed to assess the potential interactions.

## Introduction

In traditional Chinese medicine, it is a common means to include more than two drugs in a prescription to make the therapy more efficient. Lysionotin, a natural flavonoid, is commonly found in *Lysionotus* (Gesneriaceae) herbs such as *Lysionotus pauciflorus* Maxim (Teng et al. 2017). Previously, studies have reported several pharmacological properties of lysionotin, such as antibacterial, anti-inflammatory, antihypertensive, and free radical scavenging activities, and it has been widely used in the treatment of lung disease with other various drugs or herbs (Chen et al. 2002; Suksamrarn et al. 2003). Drug-drug interaction is the main factor that influences the pharmacokinetics and pharmaceutical effect, especially in traditional Chinese medicine (Ge 2019).

Cytochrome P450 (CYP) enzymes are membrane-bound hemoproteins that play a vital role in the biotransformation of xenobiotics, including drugs, environmental pollutants, carcinogens and endogenous substrates (Wrighton and Stevens 1992; Yan and Caldwell 2001). Additionally, the activity of CYPs is a vital factor that mediates drug-drug interaction and metabolism of a variety of drugs (Manikandan and Nagini 2018). For example, the pharmacokinetics of warfarin affected by the co-administration of glycyrrhetnic acid resulted in the increased plasma concentration of warfarin, due to the inhibitory effects of glycyrrhetnic acid on the activity of CYP3A4 (Song et al. 2020). It is necessary to investigate the effect of different drugs or herbs on the activity of CYPs, which would provide more reference for the clinical usage or co-administration of drugs. Dihydromyricetin, berberine, and pristimerin have inhibitory effects on the activity of CYPs (Chang et al. 2015; Liu et al. 2017; Hao et al. 2018). However, whether lysionotin could affect the activity of CYPs is still unclear.

Due to the various pharmacological properties of lysionotin and the characteristic of traditional Chinese medicine, it can be used with many other drugs or herbs in the clinic. The effect of lysionotin on the activity of CYPs was investigated in this study to provide more reference and guidance for the clinical combination of lysionotin and other drugs or herbs.

## Materials and methods

In this study, lysionotin was incubated with eight CYP isoforms (i.e., 1A2, 3A4, 2A6, 2E1, 2D6, 2C9, 2C19 and 2C8) in pooled human liver microsomes to investigate the interaction between lysionotin and CYPs, which can provide direct evidence for the effect of lysionotin on the activity of CYPs. *In vitro*, phenacetin (CYP1A2), testosterone (CYP3A4), coumarin (CYP2A6), chlorzoxazone (CYP2E1), dextromethorphan (CYP2D6), diclofenac (CYP2C9), *S*-mephenytoin (CYP2C19) and paclitaxel (CYP2C8) were used as probe substrates to determine the effects of lysionotin on eight CYP enzymes. In addition, enzyme kinetic studies were conducted to determine the inhibition model of lysionotin on CYP enzymes.

### Chemicals

**Figure 1.**
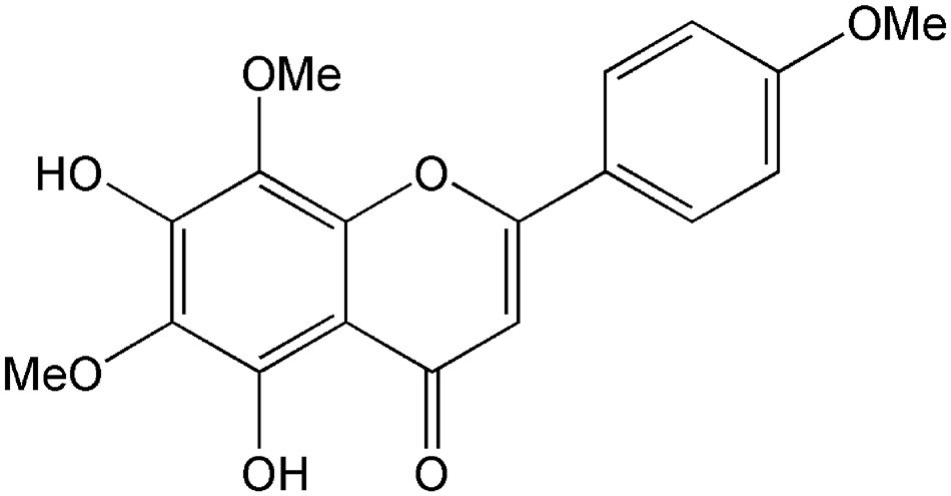
The chemical structure of lysionotin.

Lysionotin (≥98%) and testosterone (≥98%) were obtained from the National Institute for the Control of Pharmaceutical and Biological Products (Beijing, China). The chemical structure of lysionotin is shown in Figure 1. d-Glucose-6-phosphate, glucose-6-phosphate dehydrogenase, corticosterone (≥98%), NADP^+^, phenacetin (≥98%), acetaminophen (≥98%), 4-hydroxymephenytoin (≥98%), 7-hydroxycoumarin (≥98%), 4′-hydroxydiclofenac (≥98%), sulfaphenazole (≥98%), quinidine (≥98%), tranylcypromine (≥98%), chlorzoxazone (≥98%), 6-hydroxychlorzoxazone (≥98%), paclitaxel (≥98%), 6β-hydroxytestosterone (≥98%), clomethiazole (≥98%), and furafylline (≥98%) were obtained from Sigma Chemical Co. Montelukast (≥98%) was obtained from Beijing Aleznova Pharmaceutical (Beijing, China). Coumarin (≥98%), diclofenac (≥98%), dextromethorphan (≥98%), and ketoconazole (≥98%) were purchased from ICN Biomedicals. Pooled HLMs were purchased from BD Biosciences Discovery Labware. All other reagents and solvents were of analytical reagent grade.

### Assay with human liver microsomes

As shown in Table 1, to investigate the effects of lysionotin on different CYP isoforms in HLM, the following probe reactions were used, according to the previously described method (Zhang et al. 2007; Qi et al. 2013): phenacetin *O*-deethylation for CYP1A2, testosterone 6β-hydroxylation for CYP3A4, coumarin 7-hydroxylation for CYP2A6, chlorzoxazone 6-hydroxylation for CYP2E1, dextromethorphan *O*-demethylation for CYP2D6, diclofenac 4′-hydroxylation for CYP2C9, *S*-mephenytoin 4-hydroxylation for CYP2C19, and paclitaxel 6α-hydroxylation for CYP2C8. All incubations were performed in triplicate, and the mean values were utilised. The typical incubation systems contained 100 mM potassium phosphate buffer (pH 7.4), NADPH generating system (1 mM NADP^+^, 10 mM glucose-6-phosphate, 1 U/mL of glucose-6-phosphate dehydrogenase, and 4 mM MgCl_2_), the appropriate concentration of HLMs, a corresponding probe substrate and lysionotin (or positive inhibitor for different probe reactions) in a final volume of 200 μL.

**Table 1.** Isoforms tested, marker reactions, incubation conditions, and K_m_ used in the inhibition study.

CYPs	Marker reactions	Substrate concentration (μM)	Protein concentration (mg/mL)	Incubation time (min)	Estimated K_m_ (μM)
1A2	phenacetin *O*-deethylation	40	0.2	30	48
3A4	testosterone 6β-hydroxylation	50	0.5	10	53
2A6	coumarin 7-hydroxylation	1.0	0.1	10	1.5
2E1	chlorzoxazone 6-hydroxylation	120	0.4	30	126
2D6	dextromethorphan *O*-demethylation	25	0.25	20	4.8
2C9	diclofenac 4′-hydroxylation	10	0.3	10	13
2C19	*S*-Mephenytoin 4-hydroxylation	100	0.2	40	105
2C8	paclitaxel 6α-hydroxylation	10	0.5	30	16

The concentration of lysionotin was 100 μM, and the positive inhibitor concentrations were as follows: 10 μM furafylline for CYP1A2, 1 μM ketoconazole for CYP3A4, 10 μM tranylcypromine for CYP2A6, 50 μM clomethiazole for CYP2E1, 10 μM quinidine for CYP2D6, 10 μM sulfaphenazole for CYP2C9, 50 μM tranylcypromine for CYP2C19, 5 μM montelukast for CYP2C8. Probe substrates, positive inhibitors (except for dextromethorphan and quinidine, which were dissolved in water), and lysionotin were dissolved in methanol, with a final concentration of 1% (v/v), and 1% neat methanol was added to the incubations without inhibitor. The final microsomal protein concentration and incubation times for the different probe reactions are shown in Table 1. There was a 3 min preincubation period (at 37 °C) before the reaction was initiated by adding an NADPH-generating system. The reaction was terminated by adding a 100 μL acetonitrile (10% trichloroacetic acid for CYP2A6) internal standard mix, and the solution was placed on ice. The mixture was centrifuged at 12,000 rpm for 10 min, and an aliquot (50 μL) of supernatant was transferred for HPLC analysis. The instrument used in this study was Agilent 1260 series instrument with DAD and FLD detector, and the quantitative assay for the corresponding metabolites was performed as previously reported and the detection conditions are summarised in Table 2 (He et al. 2015; Lang et al. 2017; Zhang et al. 2017).

**Table 2.** HPLC analysis conditions for mentioned CYP isoforms.

CYP isoforms	Detection wavelength (nm)	Mobile phase gradient
1A2	254	Methanol (A): Phosphate buffer (pH = 3.0, 50 mmol/L) (B) = 34:66
2A6	340	Acetonitrile (A): Acetic acid (0.1%, v/v) (B) = 35:65
3A4	248	Methanol (A): Water (B) = 52:48
2C8	230	Methanol (A): Water (B) = 65:35
2C9	280	Acetonitrile (A): Phosphate buffer (pH = 7.4, 100 mmol/L) (B) = 32:68,
2C19	211	Methanol (A): Acetonitrile (B) = 69:33
2D6	244	Acetonitrile (A): Phosphate buffer (pH = 3.0, 50 mmol/L ) (B) = 25:75
2E1	281	Acetonitrile (A): Acetic acid (0.5%, v/v) (B) = 22:78

#### Enzyme inhibition and kinetic studies of lysionotin

Lysionotin (100 μM) was used to initially screen for its direct inhibitory effects towards different human CYP isoforms. For the CYP isoforms whose activities were strongly inhibited, secondary studies were performed to obtain the half inhibition concentration (IC_50_). *K*_i_ values were obtained by incubating various concentrations of different probe substrates (20–100 μM testosterone, 50–200 μM mephenytoin, 5–20 μM paclitaxel) in the presence of 0–50 μM lysionotin.

#### Time-dependent inhibition study of lysionotin

To determine whether lysionotin could inhibit the activity of CYP3A4, 2C19, and 2C8 in a time-dependent manner, lysionotin (20 μM) was pre-incubated with HLMs (1 mg/mL) in the presence of an NADPH-generating system for 30 min at 37 °C. After incubation, an aliquot (20 μL) was transferred to another incubation tube (final volume 200 μL) containing an NADPH-generating system and probe substrates whose final concentrations were approximate to *Km*. Then, further incubations were performed to measure the residual activity. After being incubated for 0, 5, 10, 15, and 30 min, the reactions were terminated by adding a 100 μL acetonitrile internal standard mix and then placed on ice; the corresponding metabolites were determined by HPLC.

To determine the *K*_I_ and *K*_inact_ values for the inactivation of CYP3A4, the incubations were conducted using higher probe substrate concentrations (approximately 4-fold *Km* values) and various concentrations of lysionotin (0–50 μM) after different preincubation times (0–30 min), with a two-step incubation scheme, as described above. The value of *K*_I_ and *K*_inact_ was calculated with the equation 1/Kobs = *K*_I_/*K*_inact_*1/[I] + 1/*K*_inact_. Where Kobs is the pseudo-first-order rate constant of inactivation at inactivated concentration [I], *K*_inact_ is the maximum inactivation rate (a theoretical value that cannot be experimentally observed), and *K*_I_ is the inactivated concentration when the rate of inactivation reaches half of *K*_inact_.

### Statistical analysis

The enzyme kinetic parameters for the probe reaction were estimated from the best fit line, using least-squares linear regression of the inverse substrate concentration versus the inverse velocity (Lineweaver-Burk plots), and the mean values were used to calculate Vmax and Km. Inhibition data from the experiments that were conducted using multiple compound concentrations were represented by Dixon plots, and inhibition constant (Ki) values were calculated using non-linear regression according to the following equation:

v=(VmaxS)/(Km(1+I/Ki+S)for  competitive  inhibition

v=(VmaxS)/[(Km+S((1+I/Ki)] for  non-competitive  inhibition

where I is the concentration of the compound, Ki is the inhibition constant, S is the concentration of the substrate, and Km is the substrate concentration at half the maximum velocity (Vmax) of the reaction. The mechanism of the inhibition was inspected using the Lineweaver-Burk plots and the enzyme inhibition models. The data comparison was performed using Student’s *t*-test and performed using IBM SPSS statistics 20 (SPSS Inc.).

## Results

### Effect of lysionotin on the activity of CYPs

**Figure 2.**
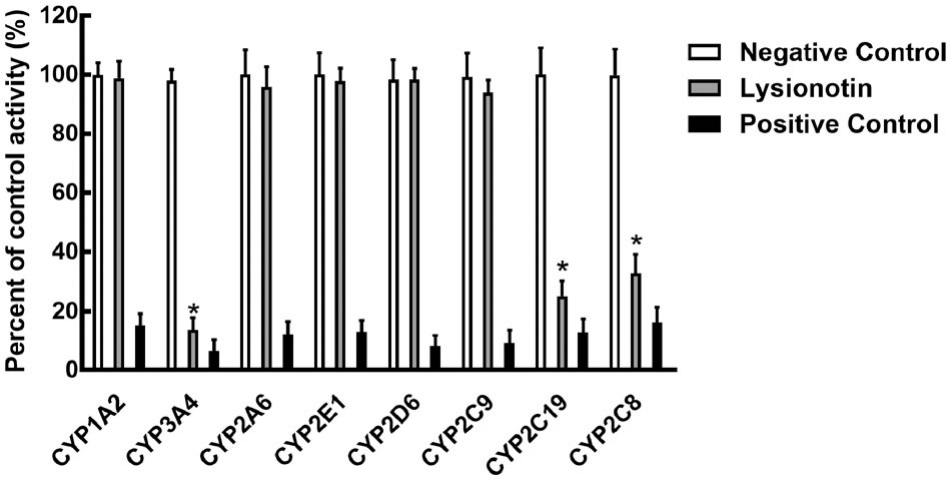
Effects of lysionotin on the activity of CYPs, including CYP1A2, 3A4, 2A6, 2E1, 2D6, 2C9, 2C19, and 2C8. *p < 0.05. Negative control: incubation without lysionotin or specific inhibitors. Lysionotin: incubation with 100 μM lysionotin. Positive control: incubation with specific inhibitors.

**Figure 3.**
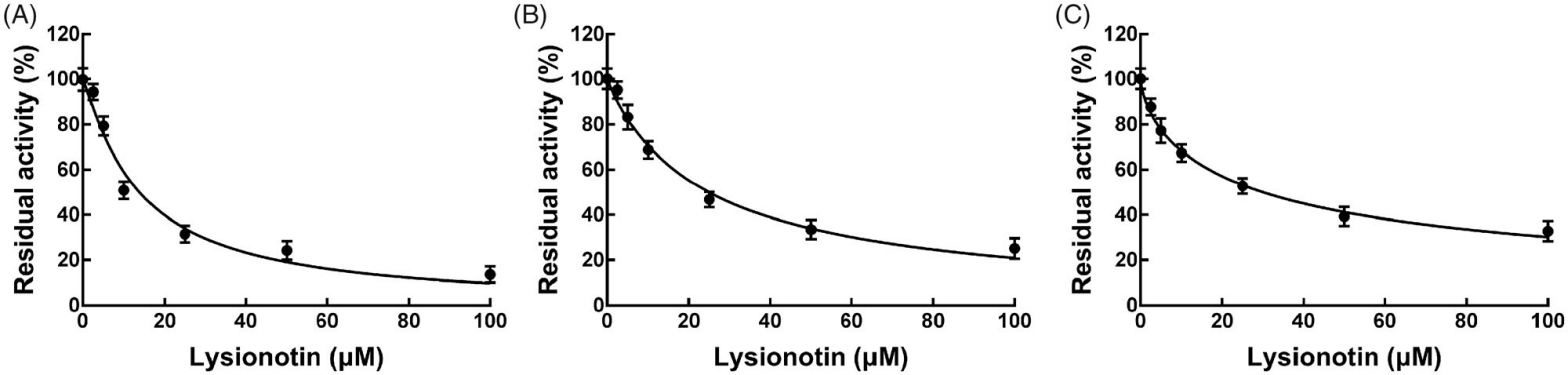
The dose-inhibition curves of lysionotin on CYP3A4 (A), 2C19 (B), and 2C8 (C).

Eight CYP isoforms were incubating with lysionotin in pooled HLMs, and the activity of CYPs was evaluated by detecting their metabolites. As shown in Figure 2, the administration of lysionotin significantly inhibited the activity of CYP3A4, 2C19, and 2C8 (*p* < 0.05), while other isoforms were not affected (*p* > 0.05). The IC_50_ values of CYP3A4, 2C19, and 2C8 were obtained by dose-inhibition curves shown in Figure 3. The activity of CYP3A4 was decreased by 86.33% with the IC_50_ value of 13.85 μM, and the activity of CYP2C19 and 2C8 were decreased by 74.86 and 67.22% by lysionotin with the IC_50_ value of 24.95 and 30.05 μM, respectively.

**Figure 4.**
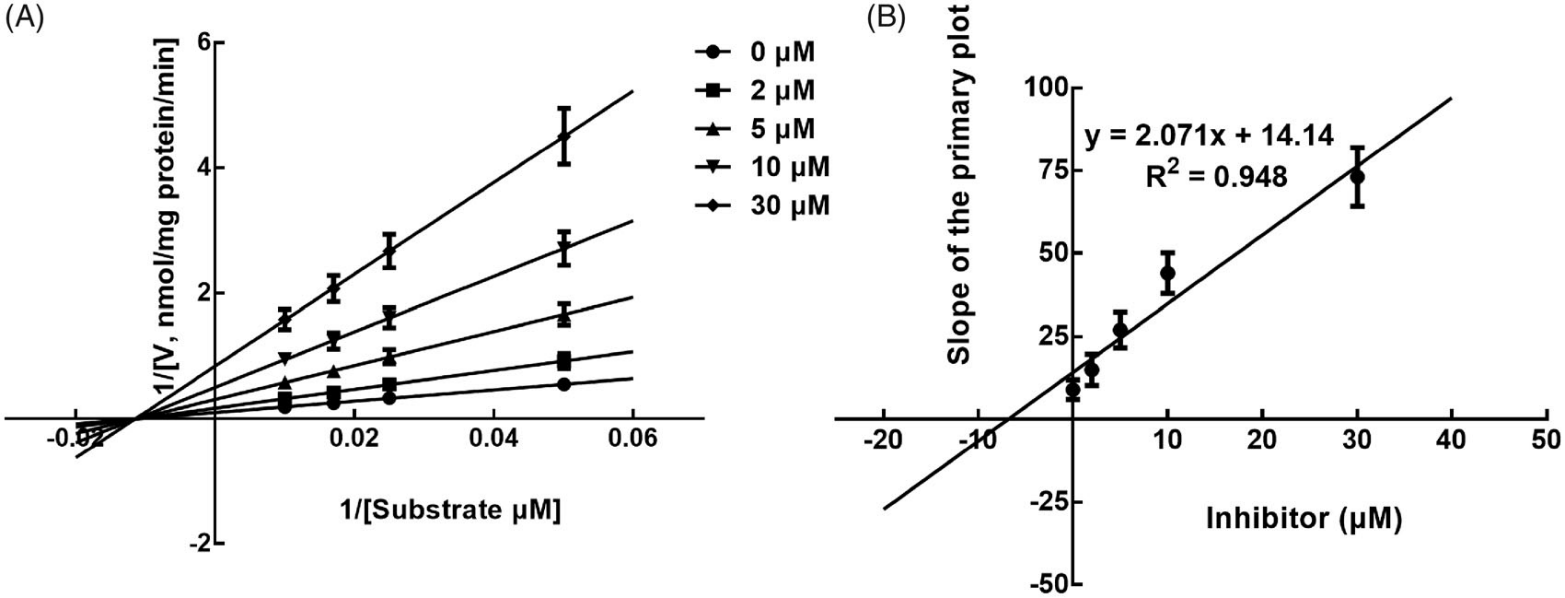
Lineweaver-Burk plots (A) and the secondary plot for *Ki* (B) of inhibition of lysionotin on CYP3A4 catalysed reactions (testosterone 6β-hydroxylation) in pooled HLM. Data were obtained from a 30 min incubation with testosterone (20–100 μM) in the absence or presence of lysionotin (0–30 μM). All data represent the mean of the incubations (performed in triplicate).

**Figure 5.**
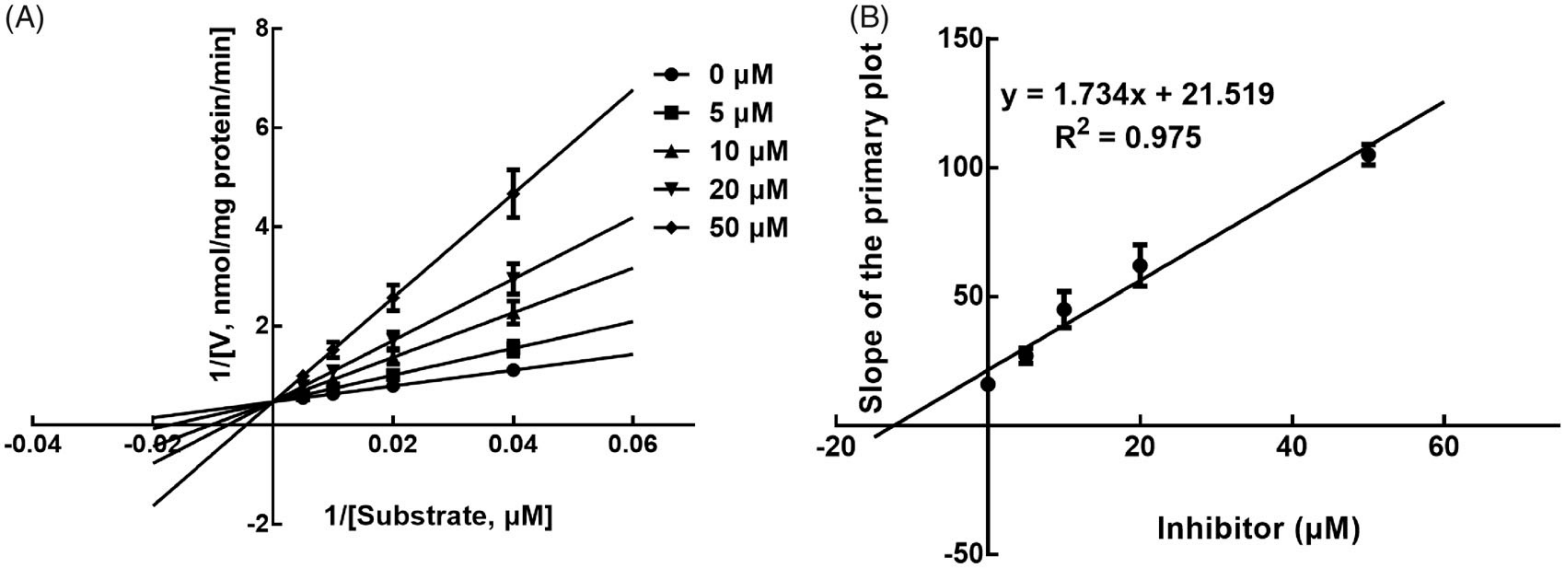
Lineweaver-Burk plots (A) and the secondary plot for *K*i (B) of inhibition of lysionotin on CYP2C19 catalysed reactions (*S*-mephenytoin 4-hydroxylation) in pooled HLM. Data were obtained from a 30 min incubation with mephenytoin (50–200 μM) in the absence or presence of lysionotin (0–50 μM). All data represent the mean of the incubations (performed in triplicate).

**Figure 6.**
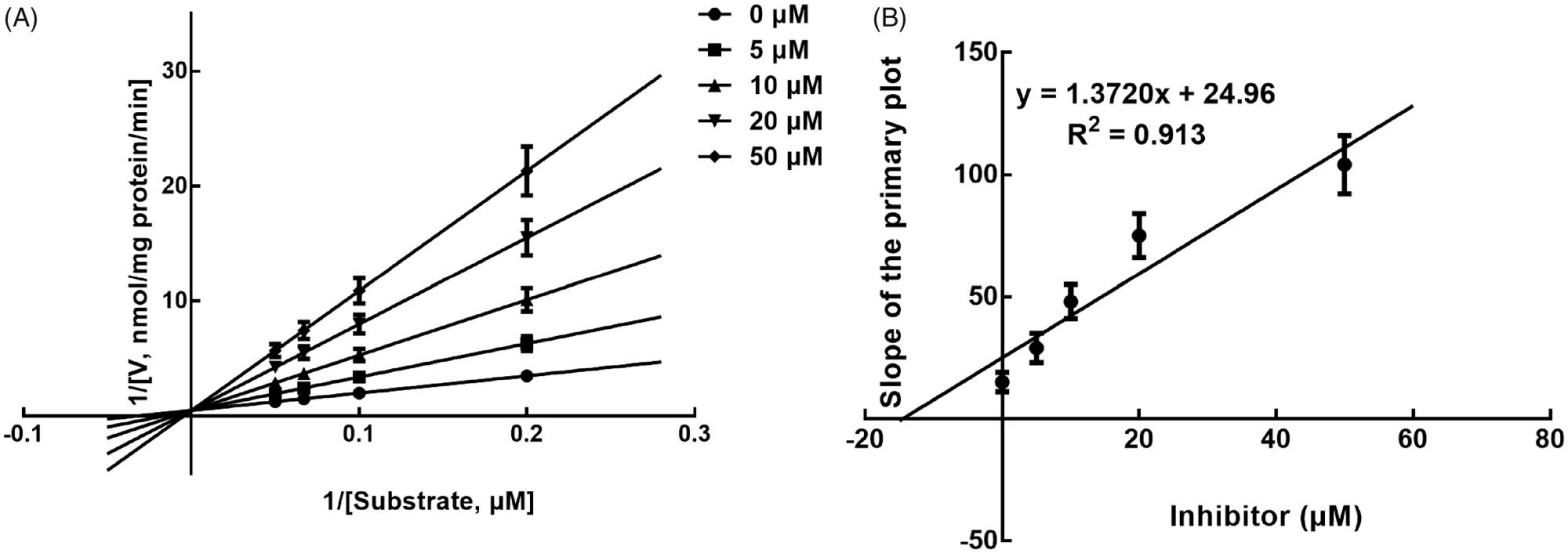
Lineweaver-Burk plots (A) and the secondary plot for *Ki* (B) of inhibition of lysionotin on CYP2C8 catalysed reactions (paclitaxel 6α-hydroxylation) in pooled HLM. Data were obtained from a 30 min incubation with paclitaxel (5–20 μM) in the absence or presence of lysionotin (0–50 μM). All data represent the mean of the incubations (performed in triplicate).

Next, the inhibition model was further investigated by fitting with Lineweaver-Burk plots. The inhibition of CYP3A4 was fitted to be non-competitive with the *Ki* value of 6.83 μM (Figure 4). On the other hand, lysionotin was found to inhibit the activity of CYP2C19 and 2C8 competitively, with *Ki* values of 12.41 and 14.51 μM, respectively (Figures 5 and 6).

### Time-dependent study

**Figure 7.**
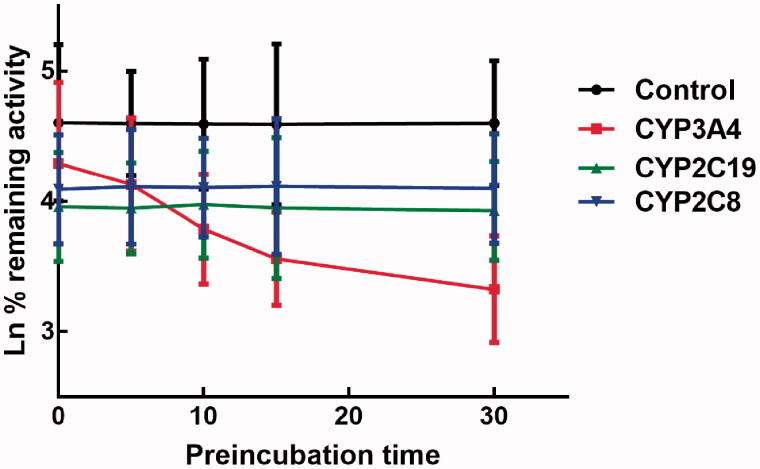
Effect of incubation time on the inhibition of CYP3A4, 2C19, and 2C8.

**Figure 8.**
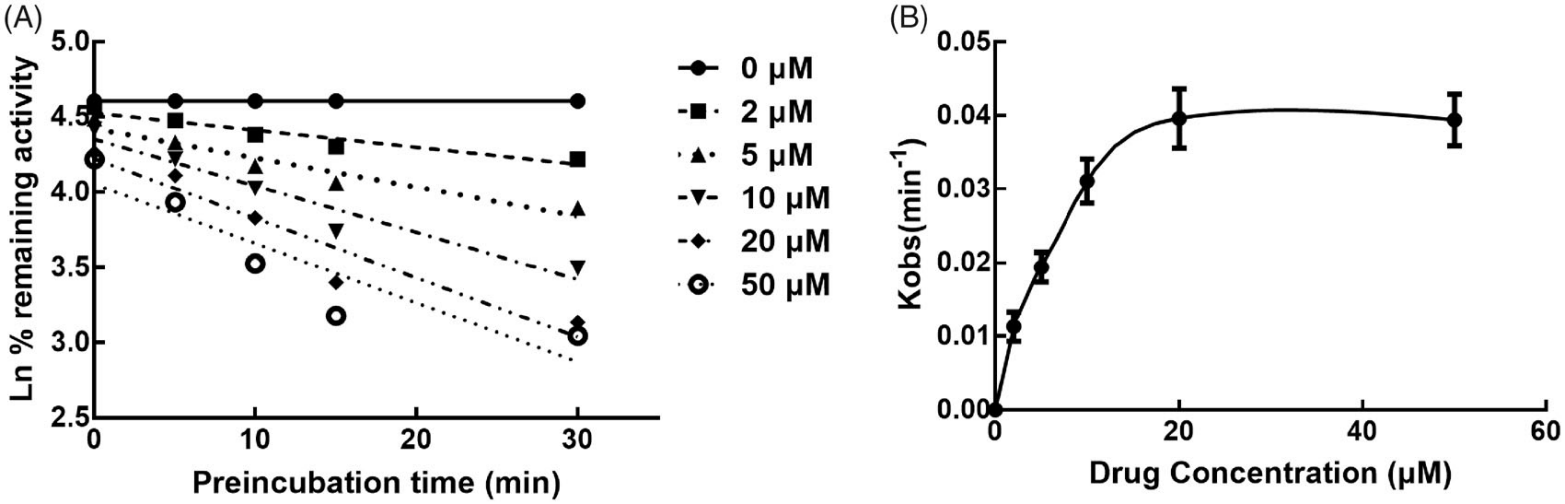
Time and concentration-inactivation of microsomal CYP3A4 activity by lysionotin in the presence of NADPH. The initial rate constant of inactivation of CYP3A4 by each concentration (Kobs) was determined through linear regression analysis of the natural logarithm of the percentage of remaining activity versus pre-incubation time (A). The *K*_I_ and *K*_inact_ values were determined through non-linear analysis of the Kobs versus the pachymic acid concentration (B).

The effect of incubation time on the inhibition of CYPs by lysionotin was investigated. It was found that the inhibition of CYP3A4 increased with the incubation time, while the inhibition of CYP2C19 and 2C8 was relatively stable, which indicated the time-dependent characteristic of the inhibition of CYP3A4 (Figure 7). Moreover, for the time-dependent inhibition of CYP3A4, the value of *K*_I_ and *K*_inact_ was further assessed. The value of *K*_I_*/K*_inact_ was obtained as 6.618/0.048 min/μM, which suggested that about 4.8% CYP3A4 was inactivated every minute during incubating with per lysionotin (Figure 8).

## Discussion

CYPs are key factors that play roles in the pharmacokinetics of various drugs (Ouyang et al. 2016; Xiao et al. 2016; Chen et al. 2017). In traditional Chinese medicine, co-administration of more than two types of drugs is very common, which can make the therapy more efficient and precise. In the co-administration of different drugs, potential drug-drug interaction may induce due to the effects that CYPs suffered. For example, atorvastatin and amlodipine are two drugs that commonly used together for the treatment of hypertension with coronary heart disease, the pharmacokinetics of amlodipine was affected by the administration of atorvastatin due to the inhibitory effect of atorvastatin on the activity of CYP3A4 (Yang et al. 2020). Therefore, it is necessary for the clinical application of lysionotin to investigate its effect on the activity of CYPs.

The *in vitro* inhibitory effect of lysionotin on the activity of CYP3A4, 2C19, and 2C8 was found in pooled HLMs in this study. Lysionotin acted as a non-competitive inhibitor of CYP3A4 in a concentration-dependent and time-dependent manner, which indicated the potential drug-drug interaction between lysionotin and drugs metabolised by CYP3A4. Moreover, it also suggested that the incubation time was an important factor during the interaction between lysionotin and CYP3A4. Therefore, the clinical administration of lysionotin should be careful and pay attention to the time of drug administration. For the inhibition of CYP2C19 and 2C8, it was performed in a competitive manner and showed no significantly time-dependent manner, which indicated that the co-administration of lysionotin with drugs metabolised by CYP2C19 and 2C8 should be avoided in the clinic.

CYP3A4 is a major member of the CYP3A family, which is involved in the liver and intestine metabolism of more than 40% of marketed drugs and pharmaceuticals and catalyses the transformation of various substrates (Denisov et al. 2019). The inhibition of CYP3A4 could directly inhibit the metabolism of its substrates, such as warfarin, puerarin, and oridonin, which is the main factor that induce drug-drug interaction (Liu et al. 2019a, 2019b; Song et al. 2020). The inhibitory effect of lysionotin on the activity of CYP3A4 indicated a potential drug-drug interaction, but it needs further *in vivo* experiments to evaluate the risk of this kind of interaction. For the CYP2C family, CYP2C19 and CYP2C8 are two major subtypes, which play vital roles in the pharmacokinetics of a number of drugs (Hicks et al. 2015). Lysionotin inhibited the activity of CYP2C19 and 2C8 in a competitive manner might be a result of the similar structure between lysionotin and their substrates. Additionally, the inhibition of CYP2C19 and 2C8 also suggested the potential effect of lysionotin on the pharmacokinetics of drugs metabolised by CYP2C19 and 2C8.

This is an *in vitro* study that investigated the interaction between lysionotin and CYPs in HLMs. The intestinal exposure of lysionotin could also interact with intestinal CYPs, which can induce adverse drug-drug interaction (Galetin et al. 2008; Zhu and Zhang 2012). Therefore, the effect of lysionotin on the activity of intestinal CYPs should be investigated in more detail in further studies.

In fact, the present study is an *in vitro* study, which cannot totally represent the situation and interaction *in vivo*. Previous study focussed on the pharmacokinetics of oral administration of 50 mg/kg lysionotin reported the *C_max_* of lysionotin in rat plasma was 58.4 ± 10.3 ng/mL, which is much lower than the obtained IC_50_ value of lysionotin (Liu et al. 2013). Therefore, the drug-drug interaction and the *in vivo* effect of lysionotin need further investigation for verification. In addition, many other factors can influence the drug-drug interaction and the pharmacokinetics of drugs. For example, pterostilbene supplements induce drug-drug interaction through inhibiting UDP-glucuronosyltransferases (UGT) 1A9 enzymes (Jiang et al. 2020). The inhibition of UGT also provides more risk of many other drugs, such as chlorophenols, oxycodone, and silymarin flavonolignans (Romand et al. 2017; Mano et al. 2018; Yang et al. 2018; Vrba et al. 2020). The specific effect of lysionotin on the activity of UGTs needs further studies. On the other hand, *P-gp* plays a vital role in the transport of various drugs, its activity is an important factor that mediates the potential drug-drug interaction. Therefore, it is also necessary to explore the interaction between lysionotin and *P-gp*.

## Conclusions

Lysionotin inhibited the activity of CYP3A4, 2C19, and 2C8 in a concentration-dependent manner. Lysionotin acted as a non-competitive inhibitor of CYP3A4 and a competitive inhibitor of CYP2C19 and 2C8. Moreover, the inhibition of CYP3A4 by lysionotin was performed in a time-dependent manner. All these results indicated the potential drug-drug interaction between lysionotin and drugs metabolised by CYP3A4, 2C19, or 2C8. Further *in vivo* studies are needed to verify these potential effects.
